# The folate receptor β as a macrophage-mediated imaging and therapeutic target in rheumatoid arthritis

**DOI:** 10.1007/s13346-018-0589-2

**Published:** 2018-10-02

**Authors:** Durga M. S. H. Chandrupatla, Carla F. M. Molthoff, Adriaan A. Lammertsma, Conny J. van der Laken, Gerrit Jansen

**Affiliations:** 1 Amsterdam Rheumatology and Immunology Center, VU University Medical Center, Vrije Universiteit Amsterdam, De Boelelaan 1117, 1081 HV Amsterdam, The Netherlands; 2 Department of Radiology and Nuclear Medicine, VU University Medical Center, De Boelelaan 1117, 1081 HV Amsterdam, The Netherlands

**Keywords:** Rheumatoid arthritis, Macrophages, Folate receptor, Folate-conjugated drugs, Imaging, Positron emission tomography (PET)

## Abstract

Macrophages play a key role in the pathophysiology of rheumatoid arthritis (RA). Notably, positive correlations have been reported between synovial macrophage infiltration and disease activity as well as therapy outcome in RA patients. Hence, macrophages can serve as an important target for both imaging disease activity and drug delivery in RA. Folate receptor β (FRβ) is a glycosylphosphatidyl (GPI)-anchored plasma membrane protein being expressed on myeloid cells and activated macrophages. FRβ harbors a nanomolar binding affinity for folic acid allowing this receptor to be exploited for RA disease imaging (e.g., folate-conjugated PET tracers) and therapeutic targeting (e.g., folate antagonists and folate-conjugated drugs). This review provides an overview of these emerging applications in RA by summarizing and discussing properties of FRβ, expression of FRβ in relation to macrophage polarization, FRβ-targeted in vivo imaging modalities, and FRβ-directed drug targeting.

## Rheumatoid arthritis

**Fig. 1:**
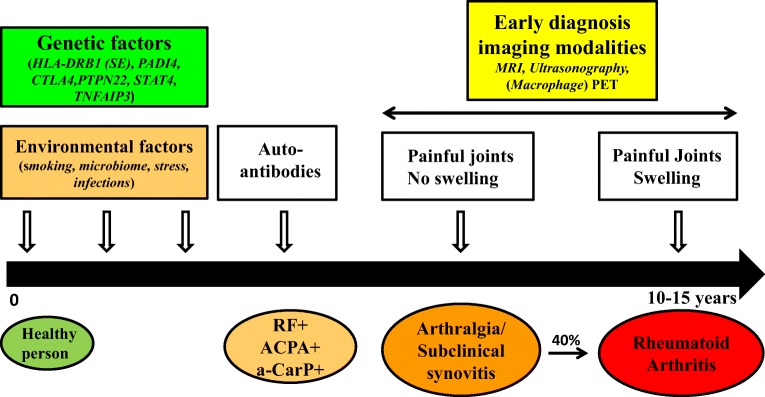
Onset of rheumatoid arthritis and positioning of macrophage imaging for early disease monitoring. Early in a time frame spanning 10–15 years, combined genetic and environmental factors can trigger in a healthy person the formation of autoantibodies which can lead to joint complaints without swelling (arthralgia). Following an unknown second hit, 40% of arthralgia patients ultimately develop RA. The subclinical stage of arthritis provides a window of opportunity early diagnosis with imaging modalities

Rheumatoid arthritis (RA) is an autoimmune disease, which affects approximately 0.5–1.0% of the world population [1]. Although the exact etiology of RA is unknown, the currently accepted hypothesis consists of two stages [2]. In genetically susceptible individuals, the first stage of development of RA consists of accelerated citrullination of proteins in extra-articular sites, e.g., due to smoking or infection, including formation of rheumatoid factor (RF), anti-citrullinated protein antibodies (ACPA), and anti-carbamylated proteins (a-CarP) [3–6]. Only 40% of ACPA-positive arthralgia individuals will eventually develop RA [7]. A second trigger seems to be needed for development of clinical disease. Up to 15 years later, the second trigger could be an unrelated episode of otherwise self-limiting synovial inflammation and associated locally induced citrullination. In the presence of pre-existing anti-citrullinated protein/peptide antibodies, this event may induce chronic synovitis evolving into clinical RA through binding of the antibodies to autoantigens in the joints [8–10] (Fig. 1).

To detect development of (subclinical) synovitis, advanced imaging techniques may have diagnostic value on top of detection of ACPA. Application of ultrasonography and MRI techniques in preclinical RA have been discussed in recent reports [11, 12], while application of positron emission tomography (PET) will be discussed in detail below. RA’s main characteristics include (chronic) inflamed synovium and joint destruction, which, when left untreated, can lead to permanent joint deformities and comorbidities, such as cardiovascular disease and osteoporosis [10]. Early identification and treatment of RA is currently recommended to prevent further joint damage and disability [13]. To this end, the European League Against Rheumatism (EULAR) guidelines indicate treatment with classical disease-modifying anti-rheumatic drugs (DMARDs) (e.g., methotrexate (MTX)), biological DMARDs (e.g., infliximab, rituximab, tocilizumab, and secukinumab), and targeted synthetic DMARDs (e.g., Janus kinase inhibitors), either as monotherapy or in combination therapy [14]. Despite this wide spectrum of potential therapeutic agents that are currently available, response to treatment usually varies between 50 and 70%. This is probably related to factors such as the heterogeneous character of RA, the stage of the disease, and the presence of anti-drug antibodies. To increase treatment efficacy and to reduce costs, monitoring tools, e.g., imaging, are needed in order to select responders and non-responders in an early phase of treatment.

## Immune cells and RA

**Fig. 2:**
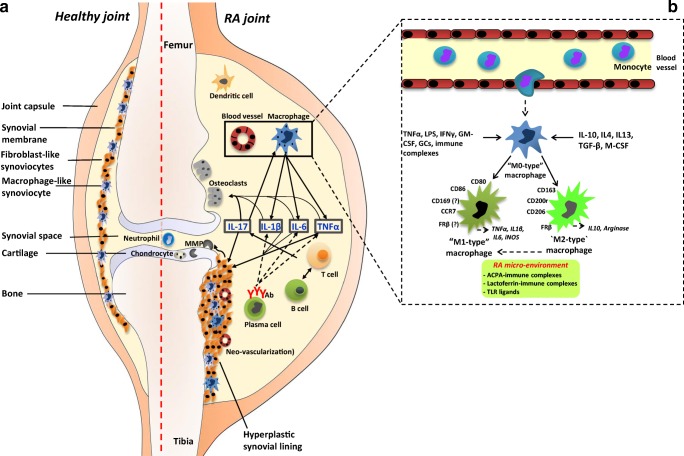
Pathogenesis of RA and the role of macrophages. **a** Schematic representation of a healthy (left) and its changes in RA (right). The healthy joint shows the synovium and synovial space between two bone ends covered with a cartilage layer. The synovial membrane separating the capsule and the synovial space consists of a thin cell layer of fibroblast-like synoviocytes (FLS) and macrophage-like synoviocytes (MLS). The RA joint features a hyperplastic synovial lining, neovascularization, and infiltration of various types of immune cells (macrophages, T cells, B cells, antibody-producing plasma cells, dendritic cells, neutrophils). The release of pro-inflammatory cytokines (a.o. TNFα, IL-1β, IL-6, and IL-17) triggers a cascade of events, proliferation and activation of FLS, activation of osteoclasts and chondrocytes, and induction of bone and cartilage destruction (via matrix metalloproteases (MMPs)), being hallmarks of RA disease. **b***Magnification inset*: Synovial macrophages are derived from influx of monocytes which, depending on stimuli by various cytokines and immune complexes, can differentiate into macrophage subtypes called M1-type and M2-type macrophages, representing the extremes of a spectrum of pro-inflammatory and anti-inflammatory macrophages, respectively. M1- and M2-type macrophages can be distinguished by membrane marker expression and cytokine release profiles. Components of the RA synovial microenvironment can alter macrophage polarization

In RA, the inflamed synovium harbors several immune cell types, especially B and T lymphocytes, dendritic cells, neutrophils, and macrophages [8–10] (Fig. 2a). As dominant producers of tumor necrosis factor alpha (TNFα), macrophages are known to play a central role in RA disease progression [15–19], macrophage production of IL1β, IL-6, and TNFα mediates proliferation and activation of fibroblast-like synoviocytes [20]. These promote formation and activation of osteoclasts and chondrocytes, which drive bone and cartilage destruction [8–10, 18, 20], being hallmarks of RA disease (Fig. 2a). Cytokine networks involving a.o. IL15, IL17, IL18, IL21, IL23, and IFNγ mediate interactions among macrophages and B cells, T cells, and dendritic cells to induce pro-inflammatory effects (reviewed in [8, 9, 21, 22]). For example, IL17 release by T cells triggers activation of synovial fibroblasts and osteoclasts [8, 21], whereas B cells/plasma cells primarily release autoantibodies such as rheumatoid factor and ACPAs to promote T cell activation [23, 24]. Macrophages in inflamed synovium are thought to be mainly derived from influx of circulating monocytes [16, 17] (Fig. 2b). Following differentiation of monocytes into macrophages, various cytokines and immune complexes can skew them in subcategories designed M1-type (pro-inflammatory) and M2-type (anti-inflammatory) macrophages, featuring characteristic cluster of differentiation (CD) membrane marker expression and release of cytokines, chemokines, and degrading enzymes [18, 19] (Fig. 2b). M1-type and M2-type macrophages do not represent static states as in an RA synovial microenvironment; M2-type macrophages can acquire M1-type properties of producing pro-inflammatory cytokines like TNFα, IL1β, and IL-6 [15–27]. Folate receptor β (FRβ) has been identified as an emerging macrophage marker. FRβ properties and clinical exploitation will be discussed in more detail in the following sections. Together, given the prominent role of macrophages in RA pathophysiology, their non-invasive visualization can hold promise for early RA disease monitoring.

## Macrophage PET imaging in RA

In RA, synovial macrophage infiltration is a hallmark of the disease, reflecting disease activity in early and established stages, being a sensitive biomarker for assessment of response to therapy [28–30]. Therefore, macrophage imaging could serve as an important clinical and diagnostic tool as well as a tool for guiding therapy in RA. Positron emission tomography (PET) is a non-invasive, in vivo imaging modality, with high sensitivity to detect active arthritis both at early or advanced stages of RA [31, 32]. It also has the ability to quantify tracer uptake, which is essential for intervention studies, i.e., for monitoring disease activity and therapy response in the whole body [33–36]. While ultrasound and MRI cover mostly detection of anatomical changes in synovial tissue [37], PET imaging allows for quantitative detection and monitoring of molecular targets. Various PET tracers have been developed to image RA. Initial macrophage-directed PET studies used [^18^F]FDG (measuring glucose metabolism in inflammatory sites) to visualize inflamed RA joints with results corresponding to clinical findings, thus providing evidence for the usefulness of PET in detecting synovitis [38–40]. This tracer showed high sensitivity, but low specificity for arthritis imaging [38]. Subsequently, PET studies were extended by using more macrophage-specific tracers (Table 1).

**Table 1:** PET tracers for macrophage imaging in rheumatoid arthritis

Name	PET isotope	Half-life (min)	Binding target	Use	Reference
FDG	18F	110	Glucose transporter	Glucose metabolism	[39, 40]
(R)-PK11195	11C	20	TSPO	Neuro-inflammation/RA	[41–44]
DPA713	11C	20	TSPO	Neuro-inflammation/RA	[42, 45]
DPA714	18F	110	TSPO	Neuro-inflammation/RA	[42, 46, 47]
PEG-Folate receptor	18F	110	Folate receptor	RA, arthrosclerosis	[48–50]

The first class of potential macrophage tracers was targeted towards the 18-kDa translocator protein (TSPO, formerly known as peripheral benzodiazepine receptor), an outer mitochondrial membrane protein that is upregulated in activated macrophages [51, 52]. *(R)*-[^11^C]PK11195 is the prototypical TSPO tracer that was employed in preclinical RA models [41, 42, 53–56] after successful application for imaging of activated microglia in neuroinflammatory diseases (reviewed in [57, 58]. In a clinical setting, significantly higher *(R)*-[^11^C]PK11195 uptake was observed in severely inflamed joints of RA patients than in moderately or mildly inflamed joints, which correlated with the extent of macrophage infiltration in excised synovial tissue [43]. In addition, subclinical disease activity could be shown when contralateral uninflamed knee joints of RA were compared with non-inflamed joints of healthy controls [43]. However, *(R)*-[^11^C]PK11195 showed limitations in detecting subclinical synovitis in RA. In particular, considerable background uptake was seen in periarticular tissue both in a rat model of arthritis [48] and in RA patients [35]. To overcome these limitations, a second generation of TSPO tracers was developed, with [^11^C]DPA713 and [^18^F]DPA714 [50, 51] having been evaluated in preclinical RA models [42, 59]. Herein, both [^11^C]DPA713 and [^18^F]DPA714 were superior to *(R)*-[^11^C]PK11195, but this still needs to be confirmed in a clinical setting.

In search for novel macrophage PET tracers in RA, macrophage markers identified on activated microglia can be helpful, e.g., CB2R and A2AR (G protein-coupled receptors), P2X7R (purinergic ion channel receptor), or matrix metalloproteinases [60].

The focus of the present review is on another emerging (activated) macrophage marker, i.e., the folate receptor β (FRβ), which potentially could also be exploited for imaging and therapeutic targeting purposes in RA [61, 62].

## Folate receptors (general properties)

Folate receptors (FR) belong to a family of two other proteins, i.e., reduced folate carrier (RFC) and proton-coupled folate transporter (PCFT). RFC and PCFT have an established function in membrane transport/internalization of folates required for a variety of biosynthetic reactions and DNA synthesis [63–66] (Table 2).

**Table 2:** Overview and expression profiling and transport kinetic features of folate transporters

Cellular (anti) folate uptake systems			
	PCFT (proton-coupled folate transporter)	RFC (reduced folate carrier)	FR (folate receptor α,β,γ isoform)
Membrane orientation	Transmembrane	Transmembrane	GPI - anchored
Localization	Enterocytes	Immune cells Tumor cells	Kidney (FRα) Tumor cells (FRα) Myeloid cells/activated Macrophages (FRβ) Hematopoietic cells (FRγ, soluble, secreted form)
pH optimum	5.0–5.5	7.2–8.0	7.4–8.0
Affinity folic acid	Km 1–5 μM	Km 200–400 μM	Kd 0.1–1 nM
Affinity 5-methyl-THF	Km 2–10 μM	Km 1–5 μM	Kd 5–10 nM
Affinity MTX	Km 2–10 μM	Km 2–10 μM	Kd 50–100 nM

FR, RFC, and PCFT differ in membrane orientation, folate substrate affinity, pH optimum, and tissue distribution [63, 66–68] (Table 2). While RFC and PCFT are transmembrane carrier proteins, FR is anchored to the plasma membrane via a glycosylphosphatidylinositol (GPI) anchor [69]. At least 3 isoforms of FR exist, FRα, FRβ, and FRγ, of which the latter is a soluble secreted form because it lacks a GPI-anchoring signal [70]. FRα and FRβ display high binding affinity for folic acid (Kd 0.1–1.0 nM), but low binding affinity for the folate antagonist methotrexate (MTX) [63, 68, 71, 72]. FRs internalize their substrates via a process of receptor-mediated endocytosis [73, 74] or potocytosis [75]. FRα has a relatively broad tissue distribution profile in normal cells (e.g., kidney) and cancer cells (e.g., ovarian carcinoma cells) [76], whereas FRβ expression is restricted to hematopoietic cells of the myeloid lineage [77, 78]. In fact, FRβ is expressed on monocytes [79], activated macrophages of RA patients [80, 81], tumor-associated macrophages [82], and acute myeloid leukemia (AML) cells [83]. A number of substances have been reported to upregulate FRβ expression, e.g., retinoic acid [84] and curcumin [85], whereas a pluripotent growth factor like activin A downregulates FRβ expression [86].

Given the fact that RFC is constitutively expressed on immune cells [87, 88], including macrophages [86, 89], and exhibits a much greater folate transport capacity than FRβ [68, 81], it is still an unresolved issue whether the primary function of FRβ in macrophages is folate transport rather than other homeostatic or immune-regulatory functions. In rapidly proliferating cancer cells, folate transporters (Table 2) facilitate folate uptake to promote DNA synthesis [66–68]. However, in inflamed RA synovium, increased numbers of macrophages are mainly derived from influx of circulating monocytes (Fig. 2b) following enhanced myelopoiesis [16]. Moreover, RA synovium macrophages display only modest cell proliferation [90, 91], thus suggesting a role for FRβ in folate uptake for macrophage proliferation may not be of primary importance. In this regard, alternative functions for FRβ have been suggested, although they still lack experimental evidence: (a) delivery of folates for biopterin metabolism, which facilitates reactive oxygen species (ROS) production in macrophages [92]; (b) FRβ-mediated scavenging of folates from sites of inflammation to deprive pathogens from nutrients [80]; or (c) involvement in signaling processes consistent with the notion that FR, as GPI-anchored protein, is localized in specialized cholesterol-rich membrane invaginations called caveolae, which harbor multiple proteins involved in signaling processes [63, 66]. With respect to the latter, a recent study reported that FRβ on macrophages had a functional interaction with CD11/CD18 to regulate cellular adhesion to collagen [93].

Beyond RA synovium, FRβ expression has been identified on macrophages in inflamed atherosclerotic lesions [94–97], accounting for cardiovascular comorbidities in RA, and tumor-associated macrophages [82, 98–100], thus underscoring that FRβ plays a role on macrophages regulating inflammatory processes. Lastly, in mice, FRβ expression has been noted on LyC6 myeloid-derived suppressor cells (MDSC), a myeloid subset capable of suppressing T cell activity [101]. So far, expression of FRβ on human MDSC counterparts has not been examined.

## Role of folate receptor β in rheumatoid arthritis

Consistent with FRβ being expressed in hematopoietic cells of the myeloid lineage [77, 78], peripheral blood monocytes (PBMs) from healthy donors and RA patients express FRβ. Based on their CD14/CD16 expression, 3 subclasses of PBMs were identified, classical (CD14^+^/CD16^−^), non-classical (CD14^−^/CD16^+^), and intermediate (CD14^+^/CD16^+^) monocytes, of which the pro-inflammatory classical monocytes expressed FRβ and were capable of binding folate-linked molecules [79]. This finding provides a rationale for targeting pro-inflammatory FRβ^+^ monocytes to suppress their infiltration into sites of inflammation, e.g., RA synovium [79].

FRβ-positive macrophages were originally identified in RA synovial fluid and assigned a functional role in methotrexate transport [102]. A study by van der Heijden et al. [81] showed that FRβ mRNA expression in synovial fluid macrophages and synovial tissue from RA patients was two orders of magnitude higher than that of T cells from the same patient. Immunohistochemical evaluation of synovial biopsies from RA patients confirmed strong FRβ staining of CD68-positive macrophages both in synovial lining and sublining [81]. Importantly, a study by Xia et al. [80] revealed that especially activated macrophages rather than quiescent macrophages, in RA synovial fluid, had high FRβ expression and concomitant folate conjugate binding activity.

Macrophage FRβ expression is not only restricted to RA, but has also been reported in other arthritis-related diseases. In temporal artery biopsies of giant cell arteritis patients, severe inflammation coincided with FRβ-positive macrophages in the adventitia [103]. In two murine models of systemic lupus erythematosis, the number of FRβ-positive macrophages correlated with disease activity [104]. Also, in two experimental models of autoimmune uveitis and autoimmune encephalomyelitis in rats, FRβ-positive macrophages were detected at local and systemic sites (e.g., peritoneal cavity) of inflammation [105]. Lastly, several studies reported the presence of FRβ on macrophages in knee sections of osteoarthritis patients [106, 107].

## Folate receptor β and macrophage polarization

Macrophage heterogeneity is a common feature in RA-inflamed synovial tissue [16–19]. Microenvironmental factors may affect both activation status and skewing of macrophages into various subsets with distinct immunophenotypes and specialized immune-regulatory and homeostatic functions. Polarization of macrophages covers the broad spectrum from pro-inflammatory to anti-inflammatory macrophages, which have been designated “M1-type” (classical activation, pro-inflammatory) macrophages and “M2-type” (alternatively activated, anti-inflammatory) macrophages, respectively [108]. Whereas M1- and M2-type macrophages represent the extremes of polarization, macrophages harbor plasticity of skewing in either direction. There are many markers that may help to differentiate M1/M2 macrophages. M1 macrophages are involved in tumor inhibition and are resistant to pathogens, whereas M2 macrophages promote tumor growth and have immunoregulatory properties [109]. Classical activation stimuli for M1-type macrophages include IFNγ, LPS, and GM-CSF; those for M2-type macrophages include M-CSF, IL-4, IL-10, IL13, glucocorticoids, and immune complexes [110, 111]. Immunophenotypically, M1-stimulated macrophages display increased cell surface expression of CD80 (provides a costimulatory signal necessary for T cell activation and survival) and CD64 (Fc-gamma receptor 1, FcγRI), while M2-stimulated macrophages have increased expression of CD163 (hemoglobin scavenger receptor), CD206 (mannose receptor), CD200R (orexin receptor 2), and CD32 (FcγRIIa) [112]. CD68 is acknowledged as one of the most common markers for identifying human macrophages [112], although its expression can also be detected on fibroblasts [113]. CD169 (Siglec-1) is a macrophage marker that is implicated in immune tolerance and antigen presentation [114]. Although CD169 has been found on activated macrophages in inflammatory diseases [115, 116], its function in RA is still unknown.

During the past decade, several studies have explored FRβ expression in the context of macrophage polarization. Initially, studies from Puig-Kroger et al. [117] showed that FRβ was preferentially expressed on M2-type macrophages following in vitro skewing of monocytes with M-CSF compared with M1-type macrophages with GM-CSF. Moreover, RA synovial fluid macrophages showed an activin A-dependent skewing to pro-inflammatory M1 macrophages and reduced expression of FRβ [118]. In synovial tissue of osteoarthritis patients, however, FRβ expression was not exclusively observed on either M1- or M2-type macrophages [119]. Some recent studies add complexity to this issue by reporting that M-CSF-polarized FRβ-expressing M2 macrophages demonstrated a high pro-inflammatory response to TLR ligands and complex IgG and/or autoantibodies to citrullinated protein immune complexes (ACPA-IC) as commonly present in RA [25, 26]. Together, these data suggest that FRβ is differentially expressed on in vitro M-CSF skewed M2-type monocyte-derived macrophages, which is in line with FRβ expression on tumor-associated macrophages [82, 99, 100]. However, in RA (and OA) synovium, inflammatory conditions alter macrophage phenotypes along with FRβ expression (Fig. 2b).

## Imaging folate receptor β in rheumatoid arthritis

The high binding affinity of folate receptors for folic acid has been exploited for the design of multiple imaging agents [120] to either detect FRα expression in tumors [121, 122] and FRβ-expressing macrophages in RA [62, 123]. Subsequently, macrophage FRβ imaging has also been applied in macrophage implicated inflammation-related diseases, e.g., asthma [124–126] and cardiovascular diseases [94, 97]. The first folate macrophage imaging study in rats with adjuvant-induced arthritis was performed using [^99m^Tc]folic acid to generate the single-photon emitting tracer [^99m^Tc]EC20, which enabled visualization of arthritic joints in a rat model [127]. Isolated macrophages from the arthritic rats also showed high FR binding capacity for folate-FITC [127]. Subsequently, [^99m^Tc]EC20 was successfully used to assess disease activity in RA patients with established disease [128, 129] as well as OA patients [107]. In RA patients, the [^99m^Tc]EC20 distribution corresponded with clinical predictors of disease activity [128]. Notably, in a subset of RA patients, [^99m^Tc]EC20 scans detected actively involved joints more accurately than clinical assessments of arthritis [128].

Further development of folate imaging agents also focused on PET tracers, which could be used for detection of (sub)clinical arthritis as well as for more accurate therapy monitoring. To this end, a folate PET tracer, [^18^F]-fluoro-PEG-folate, was synthesized in a two-step procedure and evaluated in an antigen-induced arthritis model in rats [48]. Uptake of [^18^F]-fluoro-PEG-folate was significantly higher in arthritic than in non-inflamed control knees, and also arthritic knee to bone and arthritic knee to blood ratios were higher for [^18^F]-fluoro-PEG-folate than *(R)*-[^11^C]PK11195 [48]. In addition, using [^18^F]-fluoro-PEG-folate PET, it was possible to monitor therapeutic effects of MTX in arthritic rats [49] and to monitor systemic inflammatory effects in an arthritic rat model [50]. Based on these encouraging preclinical results, [^18^F]-fluoro-PEG-folate was taken to a clinical setting in which this tracer could readily visualize arthritic joints in RA patients [130]. Recently, a novel folate-based PET tracer was synthesized in a faster (< 1 h) one-step procedure, i.e., [^18^F]-folate-PEG-NOTA-Al [131], which warrants further (pre)clinical evaluation.

Next to folate PET imaging agents, recent progress has been made in the development of folate conjugates of (near infrared) fluorescent probes that can be used for fluorescent and optical imaging purposes [58, 132, 133]. Thus far, these approaches have mostly been applied in a cancer research setting for fluorescence-guided surgery of FRα-positive tumors [134] or macrophage FRβ expression in tumors [135]. Recently, OTL-38, a novel near-infrared fluorescent folate-conjugated imaging agent, showed feasibility of imaging FRα-positive tumors [136]. OTL-38 was also examined in animal models of various inflammatory diseases including RA [137]. Interestingly, the uptake of OLT-38 in inflamed joints of the animals was shown to precede changes in clinical symptoms [137]. However, it should be noted that optical techniques have their limitations. Firstly, the penetrating power of near-infrared light is limited, so that only relatively superficial processes can be imaged. In other words, although imaging in small laboratory animals is possible, translation to the human is difficult and restricted to intraoperative imaging and possibly small hand/foot joints in RA. Secondly, as the amount of light collected by a probe depends on the depth of the source (e.g., tumor) within the body, quantification is very difficult and awaits further developments. Therefore, at this stage, optical imaging is less suited for monitoring quantitative follow-up of therapeutic interventions in vivo in humans.

## Therapeutic targeting of folate receptor β in rheumatoid arthritis

FRs have not only been exploited for imaging, but also for therapeutic targeting in cancer and inflammation [65, 66]. Targeting of FRα-expressing tumors has included folate-conjugated (a) radionuclides (α-emitters) for cancer treatment; (b) anti-cancer drugs; (c) nanoparticles containing either anticancer drugs, siRNAs, miRNAs, or genes; or (d) folate antagonists for which FRα has a high affinity [65, 68, 138].

For FRβ, similar targeting approaches are applicable [139]. Table 3 provides a selection of approaches that have been reported for targeting FRβ-expressing macrophages in RA and RA-related diseases as well as for FRβ-expressing tumor-associated macrophages and FRβ-expressing acute myeloid leukemia cells. Conceivably, applications in the cancer setting may be translatable to the RA setting. Table 3 describes several modalities for FRβ targeting, including folate antagonists, folate-conjugated immunotoxins, folate-conjugated drugs, folate-conjugated nanoparticles containing drugs or genetic material, and via chimeric antigen receptor (CAR) T cells. With respect to antifolates, several drugs inhibiting key enzymes in folate metabolisms, e.g., dihydrofolate reductase (DHFR), thymidylate synthase (TS), and glycinamide ribonucleotide formyltransferase (GARTFase) [87], were evaluated for FR-targeting and anti-arthritic activity in vitro or in arthritic animals. In general, FR has a low affinity for DHFR inhibitors, including MTX, as compared with TS and GARTFase inhibitors [68, 81]. Antifolates with selectivity for FRα and FRβ rather than other folate transporters (RFC or PCFT) include BGC-945 and selected GARTFase inhibitors. As illustrated in Table 3, folic acid conjugation to a variety of (anti-inflammatory) drugs, drug-containing liposomes, proteins, siRNAs, and miRNAs provided a bona fide vehicle for targeted delivery to FR-positive tumor cells and activated macrophages in different autoimmune inflammatory animal models. CAR T cell therapies with T cells transduced with a high affinity FRβ-specific single chain antibody represent a novel approach for selective targeting and lysis of FRβ-positive AML cells [166, 167]. Experimental therapeutics with anti-FRβ CAR T cells has as yet not been explored in relation to FRβ-positive macrophages targeting in auto-immune inflammatory diseases.

**Table 3:** FRβ therapeutic targeting in rheumatoid arthritis

Category	Remarks	Reference
Antifolates		
MTX	DHFR inhibitor, low FR affinity, high RFC/PCFT affinity	[102]
CH-1504	DHFR inhibitor, low FR affinity, high RFC affinity	[140]
EC0746	Aminopterin-folate conjugate DHFR inhibitor, activity in RA mouse model	[141]
EC0746	Aminopterin-folate conjugate DHFR inhibitor, activity in animal uveitis and encephalomyelitis model	[105]
BGC945	TS inhibitor, FRα/β specific	[81, 142]
ALIMTA/pemetrexed	TS inhibitor, moderate FR affinity, high RFC/PCFT affinity	[143]
LY309887	GARTFase inhibitor, high FR and RFC affinity, activity in mouse RA model	[144]
LY329201 and LY309886	GARTFase inhibitors, in vitro activity, and activity in rat RA model	[145]
Divers compounds	GARTFase inhibitors, FRβ selective, in vitro activity	[146]
Immunotoxins		
Anti-FRβ-PE38	Recombinant immunotoxin dsFv anti-FRβ-Pseudomonas endotoxin A (PE38). Reduction RA synovial macrophages and fibroblasts	[147–149]
Anti-FRβ-PE38	Targeting FRβ-positive tumor-associated macrophages in mouse glioma	[150]
Anti-FRβ-PE38	Targeting FRβ-positive macrophages mouse atherosclerotic lesions	[151]
Folate-conjugated nanoparticles		
G5 dendrimer MTX	Targeting mouse primary FRβ macrophages	[152]
Liposomes + MTX	Activity to FRβ-positive macrophages in mouse collagen-induced arthritis	[153]
Dextran-MTX	Activity to FRβ-positive macrophages in mouse collagen-induced arthritis	[154]
Liposomes + anti-inflammatory drugs	Targeting activated macrophages in inflammatory diseases	[155]
NFkB decoy	Delivery to murine macrophages	[156]
G5 dendrimers MTX	Targeting FRβ-positive tumor-associated macrophages	[157]
Liposomes + zoledronate	Targeting FRβ-positive tumor-associated macrophages	[158]
HSA-nanodrug	Targeting FRβ-positive AML cells	[159]
Liposomes + Dox	Targeting FRβ-positive AML cells	[160]
Folate drug conjugates		
FA-Everolimus (EC0565)	Targeting FRβ-positive rat macrophages	[161]
FDG-FA	Targeting FRα-positive tumors and FRβ-positive macrophages	[162]
Gene delivery (miRNA, siRNA)		
FA-liposomes +MCL1-siRNA	Delivery to activated macrophages	[163]
FA-micelles/hydrogels	Gene delivery to activated macrophages	[164]
FolamiRs	FA-conjugated microRNAs for delivery to FR-positive cells	[165]
CAR T cells		
High affinity FRβ-specific CAR T cells	For eradication FRβ-positive AML cells	[166, 167]

Although studies described in Table 3 underscore the suitability of macrophage FRβ targeting and imaging in RA models, several points may be considered to guide future research directions. One consideration relates to the choice of the RA animal model. For most anti-rheumatic drugs, it takes time to evaluate their action on arthritis activity when using synovial macrophage infiltration as a biomarker. Therefore, especially in the case of (sub)clinical arthritis, most existing animal models of RA may not be optimal from this perspective as they are either short-term acute models or models with severe bone destruction and/or poly-articular distribution [168, 169]. Instead, for (sub)clinical arthritis studies, antigen-induced arthritis models may be more suitable as they are more chronic and resemble human RA in terms of synovial macrophage infiltration and moderate systemic inflammation [44]. Also regarding animal studies, it is well documented that plasma levels of naturally circulating folates in rodents are 10-fold higher than in humans (≈ 100 nM vs 10 nM, respectively) [44, 170], which may increase competitive binding with an experimental folate-conjugated drug for FRβ. Lastly, FRβ expression and folate binding capacity is very much dependent on the activation status of macrophages [80], which may vary between animal models and stages of disease progression.

Optimal FRβ targeting will also benefit from information about receptor density, occupancy and kinetics (recycling rates), and levels of co-expression of any other folate transporters on target cells. In target cells with dual expression of RFC and FR, the first transporter is often dominant in internalizing natural folates and small molecule antifolates. FR can fully compensate for this when RFC expression/activity is low [171]. Since RFC, in contrast to FR, has a poor affinity for folic acid drug conjugates, FR is their sole route of cell entry and thus receptor density and recycling rates determine intracellular drug delivery to concentrations eliciting a therapeutic effect [74, 172].

## Conclusion

There is growing evidence that FRβ expression on activated macrophages represent an important biomarker in various autoimmune inflammatory diseases, including RA. FRβ expression in relation to macrophage polarization warrants further investigations under conditions mimicking inflamed RA synovium. FRβ holds promise as a target for imaging with various modalities including PET and optical imaging with rationally designed tracers. This will allow disease monitoring studies and, ideally, early identification of arthritis and PET-guided therapy response monitoring. With respect to therapy, FRβ serves as an excellent target for delivery of therapeutics to macrophages; these may include folate antagonist and folate-conjugated drugs.

In conclusion, FRβ expression on activated macrophages may be exploited to guide future diagnostics, targeted therapies, and therapy response monitoring in RA.

## Abbreviations

CD: cluster of differentiation
CTLA4: cytotoxic T lymphocyte antigen 4
DMARDs: disease-modifying anti-rheumatic drugs
FLS: fibroblast-like synoviocytes
FRβ: folate receptor β
GC: glucocorticoids
GPI: glycosylphosphatidylinositol
GM-CSF: granulocyte macrophage-colony-stimulating factor
HLA-DRB1: human leucocyte antigen DRB1
iNOS: inducible nitric oxide synthase
IL: interleukin
“M1-type” macrophage: pro-inflammatory macrophages
“M2-type” macrophages: anti-inflammatory macrophages
MLS: macrophage-like synoviocytes
M-CSF: macrophage-colony-stimulating factor
MTX: methotrexate
MRI: magnetic resonance imaging
NSAIDs: nonsteroidal anti-inflammatory drugs
PADI4: peptidyl arginine deaminase type 4
PET: positron emission tomography
PTPN22: protein tyrosine phosphatase, non-receptor type 22
RA: rheumatoid arthritis
SE: shared epitope
STAT4: signal transducer and activator of transcription 4
TGFβ: transforming growth factor β
TLR: toll-like receptor
TNFα: tumor necrosis factor α
TNFAIP3: TNF alpha-induced protein 3
